# Identifying biases and their potential solutions in human microbiome studies

**DOI:** 10.1186/s40168-021-01059-0

**Published:** 2021-05-18

**Authors:** Jacob T. Nearing, André M. Comeau, Morgan G. I. Langille

**Affiliations:** 1 Department of Microbiology and Immunology, Dalhousie University, Halifax, Nova Scotia Canada; 2 Integrated Microbiome Resource, Dalhousie University, Halifax, Nova Scotia Canada; 3 Department of Pharmacology, Dalhousie University, Halifax, Nova Scotia Canada

**Keywords:** Microbiome, Methodology, Bias, Metagenomics, Amplicon, Contamination

## Abstract

Advances in DNA sequencing technology have vastly improved the ability of researchers to explore the microbial inhabitants of the human body. Unfortunately, while these studies have uncovered the importance of these microbial communities to our health, they often do not result in similar findings. One possible reason for the disagreement in these results is due to the multitude of systemic biases that are introduced during sequence-based microbiome studies. These biases begin with sample collection and continue to be introduced throughout the entire experiment leading to an observed community that is significantly altered from the true underlying microbial composition. In this review, we will highlight the various steps in typical sequence-based human microbiome studies where significant bias can be introduced, and we will review the current efforts within the field that aim to reduce the impact of these biases.

**Video abstract**

**Supplementary Information:**

The online version contains supplementary material available at 10.1186/s40168-021-01059-0.

## Introduction

Recent advances in DNA sequencing technology have allowed community-wide investigation into the microbes that live on and within the human body. These communities and their cellular functions are known as the human microbiome, which has now been associated with multiple different influences on human health [1, 2]. Investigation into these microbes has led to the development of both novel therapeutics [3] and diagnostic tools [4]. However, results from different studies often do not match with previous findings [5, 6]. One possible reason for inconsistent results across studies is the high level of random and systemic bias that is introduced throughout sequenced-based human microbiome studies.

**Fig. 1:**
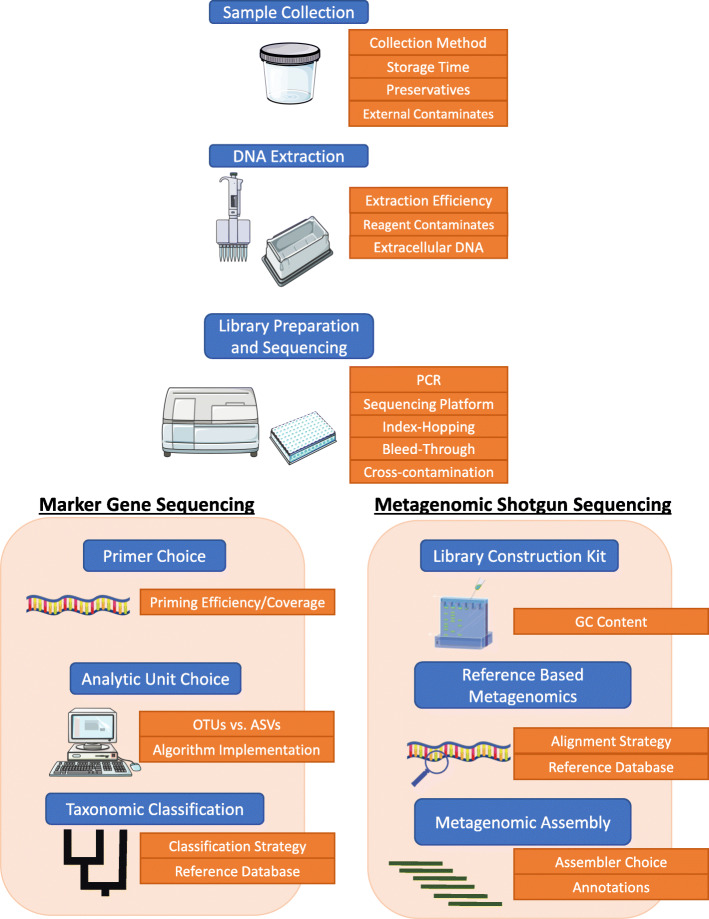
The various stages that can introduce bias in sequenced-based human microbiome studies. Each blue box represents a stage in either DNA marker gene sequencing or DNA shotgun sequencing experiments. Orange boxes represent the various areas within a stage that can result in the introduction of systemic bias. Figure created using images from Servier Medical Art (http://smart.servier.com)

Microbiome studies are particularly at fault for elevated levels of these biases due to the high sensitivity of DNA sequencing instruments and the relatively unknown underlying microbial compositions within a sample. Throughout a typical microbiome study, there are numerous areas where biases are introduced [7]. The introduction of these biases often results in distorted observations of the true underlying microbial composition contained within a sample [8]. While sometimes these biases can be subtle, they often result in significant impacts on biological conclusions. In this report, we will review each step in a typical marker gene and metagenomic shotgun sequencing microbiome study, starting with sample collection and ending with downstream bioinformatic analysis (Fig. 1). Throughout each section, we will highlight the various biases that are introduced during each step, and we will then review current approaches that have been taken to help alleviate those biases to improve our understanding of the human microbiome (Table 1).

**Table 1:** Recommended best practices during sequenced-based microbiome studies

Processing step	Recommended best practices
Sample collection	- Collect biological and technical replicates when possible. - Use the same collection device and manufacturer. - When possible, use the same collection personnel. - Use aseptic techniques during sample collection. - Make note of any variations during sample collection and include them in downstream analysis.
Sample storage	- When possible freeze samples at −80°C immediately upon collection. - Exposure of samples to room temperature conditions should be minimized. - Preservatives should be used only when freezing of samples is infeasible (e.g., self-collected human samples). - If used, all samples should be stored in the same preservative. - Length of sample storage should be noted and included in downstream analysis.
DNA extraction	- Extractions should be done using validated extraction kits or validated protocols such as those presented by the Earth Microbiome project or International Human Microbiome Standards. - All samples must be extracted with the same protocol. - Extraction batches should be noted and used as covariates in downstream analysis. - Extraction should include a mechanical lysis step (e.g., bead-beating). - Extraction should be done using aseptic techniques and a biological safety cabinet to reduce the amount of possible contamination. - A small pool of samples should be extracted during each extraction batch and sequenced to determine technical variation. - Blanks should be carried through extraction to sequencing (critical for low-biomass samples).
PCR amplification	- Use high-fidelity polymerases. - Minimize the number of required PCR cycles (preferably max. 20–25). - Obtain as uniform amplification as possible for all samples. - Primers should be chosen based on the microbes of interest and whether the work is to be compared against previous literature.
Metagenomic library construction	- Use equal amounts of template for library construction. - Avoid usage of discontinued Illumina Nextera XT kit. - Note mechanical sonication can cause minor biases toward high-GC content sequences.
Marker gene bioinformatics	- Use denoising algorithms such as Deblur, DADA2, or UNOISE. - Use validated taxonomic classification methods such as QIIME2 feature classifiers or Kraken2. - Taxonomic classifiers should be consistent between comparison studies. - Use well-curated, up-to-date, comprehensive taxonomic databases such as SILVA, RDP, or NCBI.
Metagenomic shotgun bioinformatics	- Referenced-based analysis: ◦ Use DNA-based KMER taxonomic assignment such as Kraken2 or taxMaps. ◦ Removal/filtering of low abundance taxa is recommended. ◦ Employ phylogenetic marker-gene-based strategies when examining low abundance taxa. - Metagenomic assembly: ◦ Use well-validated assembly methods such as MEGAHIT or metaSPAdes. ◦ Use validated binning methods such as DASTool, MetaWrap, or MetaBat2. - For all methods, reference databases should be well-curated and up-to-date such as: ◦ NCBI RefSeq ◦ Genome Taxonomy Database

## Sample collection and storage

Sample collection and storage are a vital part of an experiment and if not carefully planned can often introduce unaccounted bias. The way in which the sample is collected, how long the sample is stored, and what the sample is stored in can all impact the underlying observed microbial composition. All these details should be noted during sample collection and accounted for during downstream analysis to ensure consistent results across studies.

### Sample collection

The method of sample collection is particularly important to consider as different types of systematic bias can be introduced depending on the sampling environment. This has been highlighted in studies of the various areas of the human microbiome. Work on the gut microbiome has compared colon biopsy samples to both stool and rectal swab samples. In both cases, it was found that collection of samples by biopsy introduced strong biases toward the identification of specific microbes such as those that adhere to the mucosal wall of the colon [9–12]. This is not surprising given the drastically different collection methods. For example, biopsies often have higher levels of human DNA and do not account for impacts on community composition during the transit of stool in the colon [13, 14].

In contrast, multiple studies have compared the observed microbial communities from rectal swabs and fecal samples and have found similar profiles. Bassis et al. [15] compared stool samples and rectal swabs from the same patients and found no significant difference in microbial abundances between these collection methods. A further report by Reyman et al. [16] has shown similar results; however, a recent study by Jones et al. reported significant differences in both taxonomic makeup and functional pathway abundances between rectal swab and stool samples. They found that 24 of 48 families were significantly different in relative abundance between swab and stool samples through 16S rRNA gene sequencing, and a higher proportion of aerobic bacteria could be found in rectal swabs. Furthermore, they found numerous differences in levels 1 and 2 KEGG pathways through metagenomic shotgun sequencing (MGS) analysis. However, it should be noted that they did find rectal swab microbial profiles, matched stool profiles closer than biopsies [17]. Overall, these results indicate that while stool and biopsy samples reveal different microbial communities in both membership and diversity, rectal swabs may provide a viable easy-to-collect alternative to stool while keeping in mind that the proportions of aerobic genera will be elevated.

Another body site of interest that has been studied comprehensively is the human oral microbiome. Within this environment, it has been shown that while different sites of the oral cavity such as the tongue, dental plaques, and saliva accommodate unique microbial communities [18, 19], the sampling of these communities using different techniques has been shown to be comparable. Work by Fan et al. [20] and Jo et al. [21] compared human oral microbiome compositions collected through several approaches, including mouthwashes, water rinses, and unstimulated or stimulated saliva. They found no significant differences in community composition at the genus level due to the sampling method. Furthermore, work on comparing dental plaque collection methods found no differences in total DNA extracted, alpha diversity, or overall community structure between using a scaler or CytoSoft brush [22].

Several other body sites contain microbial communities that have been examined using several different collection methods. Studies that have compared different sampling techniques used in these areas have shown variable results. For example, reports have shown that skin swabs and tape-stripping result in similar family level relative abundances (Rho 0.792-0.999) [23] and no differences in alpha diversity [23, 24]. Interestingly, work by Bjerre et al. [25] comparing skin swabs and skin scrapping showed significant differences in alpha diversity between sampling techniques despite having an overlap of ~90% between the OTUs identified through each method. A similar conclusion was found between lung brushings and exhaled breath condensates where lung brushings were found to have significantly higher DNA levels and cluster separately from breath condensates when comparing overall community structure [26]. Overall, these cases highlight the fact that new collection methods should be compared with previous work to determine whether they will introduce, or conversely reduce, a significant amount of bias during downstream analysis.

Overall, the most important thing to consider during sample collection is the use of consistent collection methods within any given analysis. The same aseptic techniques should be employed during the collection of all samples. Furthermore, due to the high sensitivity of sequencing instruments, the use of the same type and manufacturer of collection devices is heavily advised, as DNA from collection devices can be introduced into samples [27]. When possible, all samples should be collected by the same individual as batch effects may be introduced due to differences in the individual collectors. This could be due to several issues including slight variations in collection technique between individuals or the individual’s own microbial flora contaminating the sample. This principle also holds true for sample preparation and sequencing. Any variation in sample collection methodology, such as those highlighted above, should be noted during collection and be included as confounded effects during data analysis.

### Study design

While the proper collection of samples is critical for any microbiome study, samples need to be collected in a manner that can be used to answer the hypothesis in question. Common study designs for microbiome studies include longitudinal, cross-sectional, and cohort designs [28]. While a thorough description of study designs is beyond the scope of this review, we have highlighted a number of factors that should be of particular importance to reduce systemic bias or confounding information within your study.

All studies should randomize samples during extraction and sequencing to ensure that technical variation is equally spread between samples. Previous work has shown that batch effects due to non-random sample preparation can lead to spurious results [29]. Furthermore, when possible, samples should be extracted using the same extraction kit lot and within the smallest time frame as possible. When this is not possible, extraction kit lot numbers and extraction dates should be included as confounding variables during data analysis. During the identification and recruitment of participants in your study, we suggest matching them on basic dietary information along with their age and BMI, although it should be noted that these choices are sample specific, as recent work on the oral microbiome has shown that the effect size of these factors is relatively small with no single factor explaining a variation larger than 2% [30]. Finally, the most important thing researchers need to do is to be consistent in the way samples are handled, and when abnormalities occur, they should be noted and included during any data analysis.

### Timing of sample storage

It often is impractical to immediately analyze fresh samples in microbiome research. As a result, many samples are preserved through storage at −80°C. It remains unclear whether freezing samples significantly affects the resulting community profiles. Work by Bahl et al. [31] compared frozen and fresh human stool samples from the same donors using quantitative PCR (qPCR) and found significant differences in the *Firmicutes* to *Bacteroidetes* ratio. However, subsequent work by Fouhy et al. [32] comparing frozen and fresh stool samples using both culture and DNA sequence-based approaches showed no significant differences in the relative abundances of bacteria at the phylum or family level. At the genus level, there was only minor evidence suggesting that the relative abundances of *Faecalibacterium* and *Leuconostoc* may be biased due to sample freezing. Taken together, these results have helped reinforce the current standard of immediately freezing samples at −80°C upon collection [33].

Research on what happens to room temperature-stored stool, which can commonly occur when freezers are not readily available, has shown promising results. Work by both Shaw et al. [34] and Bokulich et al. [35] has shown that fresh and 2-day-old stool and stool swabs have similar alpha diversity measurements and observed microbial relative abundances. However, Shaw et al. did note significant differences in weighted beta diversity measurements after 48 h of storage. Longer durations of room temperature storage, such as those sometimes seen during the shipping of self-collected stool samples, has indicated that Enterobacteriaceae and other aerobic bacteria can begin to bloom while alpha diversity measurements such as richness can decrease over time [35–37]. Fortunately, work by Amir et al. has characterized 20 different 16S rRNA gene sequences mainly belonging to Gammaproteobacteria that commonly bloom during room temperature storage [38]. Based on these identifications, they have developed bloom-filtering software that reduces taxonomic biases due to room temperature storage and have successfully implanted its use in the analysis of data from the American Gut project [36].

Not only does the length of time samples remain unpreserved need to be considered when looking at biases during microbiome studies, but so does the length of time samples are frozen. Work by Lauber et al. [39] compared multiple different short-length storage timings (3–14 days) of frozen stool and skin samples and found that samples clustered together based on their sampling source rather than the amount of time they were frozen, indicating minimal impact on overall microbial composition in the short term. Investigation into longer freezing times by Shaw et al. [34] found that all diversity metrics remained stable throughout storage with the exception of OTU richness, which slightly decreased over time. Comparing taxonomic abundances between samples stored from 2 months up to 2 years resulted in no significant differences. Overall, these findings indicated that long-term storage may introduce small biases in overall sample richness that should be taken note of during analysis, but the overall community structure and relative abundances of microbial DNA within samples remains similar for at least 2 years of storage time. Based on these results, when examining samples from longitudinal designs, researchers should be weary of any conclusions showing slight consistent reductions in microbial richness over long periods of time unless samples were sequenced upon or near the collection date.

### Sample preservatives

While the current gold standard of sample preservation is to immediately store samples at −80°C, this is not always feasible. This had led to the development of multiple different preservatives that allow samples to be stored at room temperature for extended periods of time. This is accomplished by inhibiting microbial growth within the sample while preserving microbial DNA. Currently, there are several different preservatives that can be used to store microbiome samples including RNAlater, OMNIgene preservatives, Tris-EDTA, FTA cards, and ethanol. While in many cases the use of preservatives has been shown to not impact DNA quality or yield [33], they often introduce biases toward the detection of specific microorganisms. In 2015, Choo et al. [40] compared commonly used microbiome preservatives for stool and found that each one was linked to the introduction of bias in observed community composition. Preservation in OMNIgene.GUT and RNAlater was linked with inflated microbial diversity when compared to frozen samples, and Tris-EDTA usage led to significant differences in the relative abundance of multiple taxa [40]. Additional work on biases introduced due to the use of preservatives has shown that the use of RNAlater leads to significant biases toward the detection of certain taxonomic groups [41, 42], and that Stool Nucleic Acid Collection and Preservation tubes (Norgen BioTek Corp.) can result in the over-identification of gram-negative bacteria [43]. Finally, work by Song et al. [37] has shown that the use of FTA cards results in higher levels of taxonomic diversity, but only small consistent differences in taxonomic proportions. Overall, these results indicate that the use of preservatives can have a substantial impact on the observed relative abundances of different taxonomic groups within a sample, but large scale overall community structure metrics may be comparable between some methods [44]. Since the type of preservative chosen can result in a biased observation of the true underlying microbial community, many may want to avoid their use. If freezing samples immediately is not possible, the same preservative should be used for all samples within a study and should be noted within the methodology section of the corresponding manuscripts.

### Benchmark sample/technical replicates

A possible solution that could aid in dealing with batch effects during both sample collection and downstream processing is the increased usage of technical replicates. Currently, the use of technical replicates during a microbiome study is uncommon. While sequencing facilities may initially benchmark their sequencing process on multiple mock communities, biological samples or samples reflecting a composition like those of the sample of interest are rarely sequenced twice. This is often due to budgetary constraints and the thought that a larger number of unique biological samples is a better use of resources than the creation of technical replicates, especially when they are pseudoreplicates and not true biological replicates. While there may be some truth to this argument, the use of technical replicates has numerous benefits and serves as an important tool to help identify batch effects during sample processing. It allows the researchers to identify the amount of variance in their sample preparation procedures and, as such, allows one to determine the minimal effect size that can be reliably determined as being from biological signal vs. originating from technical variation.

One possible avenue researchers could take to help with the budgetary constraints of the creation of technical replicates is to pick a small group (or one pool) of samples that will be processed throughout each batch of sample processing. By comparing the same biological sample throughout numerous sample batches, researchers can benchmark the variance in composition that they can expect between sample sequencing and preparation rounds. While in the past, mock communities have generally served this purpose, they often do not reflect the composition of the samples of interest, and, as such, a biological sample that is similar in composition to those under examination should be chosen.

## DNA extraction

After sample collection and storage, the next step in most microbiome experiments is DNA extraction. During this step, there are three main areas that can result in significant biases within a study: the differing DNA extraction efficiencies of microorganisms, the introduction of contaminant DNA, and the introduction of DNA from non-viable microorganisms. Due to these issues, DNA extraction is one of the most biased steps in sequenced-based human microbiome studies [6, 45].

### Extraction efficiencies

It is well-known that many microbes exhibit different DNA extraction efficiencies that can result in lower or higher yields of DNA. For example, both bacterial endospores and gram-positive bacteria are known to produce less DNA upon extraction compared to gram-negative bacteria [46]. These large differences due to cell wall structure can be alleviated using mechanical cell lysis techniques such as bead-beating [47]. Nonetheless, even with the inclusion of bead-beating steps, it has been suggested that extraction biases remain even between species in the same genera [6, 48, 49]. For example, despite using the same number of starting cells and accounting for genome size, Morgan et al. [48] found that two strains within *Lactococcus lactis* can exhibit different DNA extraction efficiencies. While cell wall architecture could account for many differences, it is not understood why closely related species would have different DNA extraction efficiencies. Without a better understanding of the factors underlying differential extraction efficiency, this step will remain highly biased. As such, it is recommended that future researchers identify the extraction efficiencies of health-related microbes starting from known cell counts for those organisms.

Further work in this area has also shown that the use of different DNA extraction kits can result in different DNA extraction efficiencies for taxa depending on the kit used [50]. This can result in systemic biases toward the detection of specific bacteria in microbiome studies. Early work by Carrigg et al. and Krsek et al. using denaturing gradient gel electrophoresis showed that different DNA extraction protocols used on soil and sediment samples significantly altered the observed microbial profiles [51, 52]. Furthermore, work by Costea et al. [53] compared 21 different extraction protocols and found that, while taxon abundances correlated strongly between protocols, there were significant differences between them based on various beta diversity measurements. Overall, their work along with others has shown that while technical biases due to the use of different DNA extraction kits are smaller than interindividual variation, they still posed significant issues for downstream analysis [54, 55]. Based on these studies, it is highly recommended that researchers include a mechanical lysis step during DNA extraction and either use a standardized kit [48] or well-validated protocols such as those presented by the International Human Microbiome Standards project [53].

In addition, the amount of systemic bias introduced due to differences in DNA extraction protocols has been recently linked to the level of biomass within a sample. A recent study by Davis et al. [56] has shown that biases due to choice of DNA extraction kit are stronger in low-biomass samples compared to high-biomass samples. This critically shows that comparisons of low-biomass samples should be done using the same DNA extraction protocols and that extreme caution should be taken when comparing results between studies with different DNA extraction methods.

In fact, almost all systematic bias that is introduced during sample collection and processing is amplified when working with low-biomass samples such as breast milk [57], lung samplings [58], tumor tissues [59], or placental tissue [60]. This is due to factors such as contamination or sampling procedures having a larger relative impact on community composition than they would in high-biomass samples such as human stool. As highlighted by Greathouse et al. [50], it is important to reduce all possible sources of contaminants during sample preparation and to show the existence of microbial life beyond sequencing. This could include approaches such as fluorescence in situ hybridization, microscopy, or culturing. Furthermore, it is recommended to use technical replicates during the sequencing of low-biomass samples to ensure the reproducibility of the sample composition between extractions and sequencing runs.

### Contaminant DNA

Contamination is a common issue that can be introduced during multiple steps of sequencing experiments including during sample collection, DNA extraction, and DNA sequencing. Sequencing experiments are particularly vulnerable to the introduction of contaminants due to the high sensitivity of DNA sequencing platforms. This is highlighted in their ability to sequence DNA in samples that appear DNA-free based on gel electrophoresis [61]. In a recent review of 265 microbiome studies by Hornung et al. [62], only 30% of studies were found to report using any type of negative control during sequencing experiments. Furthermore, it was unclear in many cases whether the negative control was only analyzed by gel electrophoresis or also subjected to DNA sequencing. This highlights the fact that many microbiome studies could be potentially biased due to the introduction of contaminants.

Fortunately, in many cases, such as during the analysis of fecal samples or oral washes, contamination will only account for a very small proportion of total biomass and as a result only create a minor amount of bias [63]. However, as mentioned above, contamination can be a major issue in low-biomass samples such as those collected from the human airway, brain, or placenta [63]. In some cases, such as for placental samples, the identification of microbes has been argued to be purely due to contamination itself [64–66]. This has led to studies on the “kitome” which represents the microbial inhabitants of commonly used lab reagents. These studies have revealed that while lab reagents are DNase free, they are often not DNA free [61]. Multiple different strategies have been implemented to remove contaminant DNA from lab reagents, including the use of restriction endonucleases [67, 68], UV irradiation [69], and ultrafine filtration [70]. In a recent report, Stinson et al. [71] compared low-biomass samples that were prepared using standard PCR master mixes or PCR master mixes treated with dsDNase. They found that treatment with dsDNase resulted in a significant reduction of contaminant bacterial reads within their samples. This indicates that the simple pre-treatment of PCR master mixes with dsDNase may result in improved microbiome analysis for low biomass samples. However, it still remains unclear whether any of these potential solutions can reliably remove all contaminant DNA [71] and as such most microbiome samples will contain some amount of contamination. As this remains true, it is critical for low-biomass samples to include extraction blanks that have been carried through the entire extraction and sequencing process in order to determine the level of contaminants and whether your samples contain enough reads to determine true biological effects above the “background” of the blanks.

While DNA extraction is arguably the most susceptible step to contamination issues, there is evidence that sequencing and library preparation can also be affected [72]. Index hopping has been identified as a potential source of contamination between samples during library preparation, especially on Illumina instruments that use ExAmp chemistry on patterned flow cells [73, 74]. Index hopping refers to DNA sequence barcodes being swapped between samples due to recombination events, which leads to sample cross-contamination. Furthermore, there is evidence of sample bleed-through during sequencing, which refers to DNA from previous sequencing runs being incorporated into later runs. Although we are in the process of comprehensively evaluating the effect of bleed-through, on the Illumina MiSeq, it is currently thought to contribute to 0.1% of total DNA reads per run [75].

### Post hoc contamination removal

Due to the significance of this issue, various bioinformatic techniques have been suggested as ways to identify potential contaminant sequences. In 2017, Edmonds and Williams [76] suggested that complete removal of OTUs found in negative/blank sequencing controls could be used to eliminate potential contaminant sequences. However, this is not always advisable as complete removal can lead to the loss of biologically relevant signals due to cross-contamination between samples and negative controls [77]. A second common approach for contaminant removal is the removal of sequences below a specified relative abundance or read count threshold. Nonetheless, this also does not work well in practice on low-biomass samples and in some cases will lead to the removal of rare features that are truly present [78].

In 2018, Davis et al. [78] presented a novel method, “decontam,” which attempts to identify levels of contamination rather than completely remove suspected contaminates. In this method, the authors take advantage of two different patterns of contamination through the measurement of DNA concentration levels and the sequencing of negative controls. The first pattern is that the abundance of contaminant sequences tends to be inversely proportional to the total DNA concentration in a sample, whereas true sequence abundance is independent of DNA concentration levels. This allows the authors to fit models of contamination to various sequences and remove contaminant abundances according to significant model fits. The second feature they take advantage of is the greater presence of contaminant sequences in negative controls than in real samples. While the authors show their tool is effective at controlling contamination in multiple different types of samples, their models still cannot account for cross-contamination between biological samples, nor the more significant problem of sequence bleed-through on Illumina machines.

Currently, to the best of our knowledge there are no bioinformatic methods that can deal with cross-contamination between samples in microbiome data. However, Minich et al. [72] have suggested a few steps that can be used to reduce its effect on microbiome analysis. These steps include that samples should be randomized across plates, and, when possible, single-tube extraction methods should be employed. Furthermore, low-biomass samples should not be prepared on the same plates as samples with high biomass.

Overall, while the use of reagents to remove microbial DNA may be promising, they have yet to be consistently shown as a valid approach to reduce contamination. However, researchers might think about opting for the use of a similar strategy when working with low-biomass samples. Furthermore, the removal of contaminants post hoc using bioinformatic software such as “decontam” should be included as part of the standard analysis of low-biomass samples, but will only provide minimal improvements to high biomass samples such as stool [78]. Finally, as previously mentioned, sample collection and processing should be done using aseptic techniques and when possible completed within biosafety cabinets.

### DNA from non-viable cells

One of the final bias that can be introduced during DNA extraction is the inclusion of extracellular DNA found in biofilms and dead cells. DNA included from these sources can often result in the obstruction of phylogenetic signals and the over-identification of specific microorganisms [79]. This has been shown to be particularly problematic in the study of the airway microbiome in cystic fibrosis patients due to the biofilms formed by resident bacteria [80]. However, recent work on the gut microbiome of rabbits has shown that the inclusion of dead cells in fecal samples can result in significant biases and interfere with identifying the true live microbial community within a sample [81].

Various methods have been introduced to identify the living portion of microbial communities [79]. One proposed method to distinguish viable and non-viable cells takes advantage of the fact that RNA is only actively transcribed in living cells and is degraded quickly when exposed to factors outside of the cell. Therefore, by sequencing RNA (cDNA) instead of DNA, only genetic information from the living fraction of cells within a community will be captured. Nonetheless, this method suffers from multiple weaknesses, including the lower stability of RNA prevent the use of some samples (ex: stool), a higher detection rate of non-dormant microorganisms [82], and that rRNA transcription levels and cellular growth rates are not perfectly associated [83].

A second promising approach takes advantage of cell membrane integrity and the ability to pass specific chemicals through the membranes of non-viable cells. While these techniques are commonly used in microscopy and flow cytometry, it has only recently been applied to DNA sequencing experiments [79]. Recent work by Weinmaier et al. [84] has shown that the use of propidium monoazide (PMA), a chemical that binds extracellular DNA and inhibits PCR amplification, can significantly improve the ability to identify DNA from live cells prior to sequencing. Since this initial work, multiple other studies have successfully taken advantage of PMA to characterize differences between the “live” microbiome and the total microbiome [85–87]. While this method is promising, it cannot accurately distinguish between viable and non-viable spores and archaea [84]. Further caution is also required when using this method as certain microbes such as strict anaerobes that are alive within the sampling environment upon collection will die off during sample processing. This can result in the loss of information from these groups of microbes, despite them being alive and present in the original sample.

## Library preparation and sequencing

### DNA amplification

Multiple factors can lead to biases at the PCR amplification step of microbiome studies. One of these factors is the inclusion of both inorganic ions and organic material that can interfere with PCR amplification [88]. The inclusion of these materials is usually due to inefficient DNA extraction steps in poorly optimized sampling environments. Interference by the inclusion of these inhibitors can lead to reduced PCR amplification and the inability to identify low abundance organisms within a sample.

One countermeasure to the reduced PCR amplification due to inhibitors is to increase the number of PCR cycles. Increasing PCR cycle numbers has been shown to significantly increase bacterial richness while not significantly impacting overall community structure due to the stronger detection of low abundant organisms [89]. However, this does come at the cost of reduced specificity. Work by Qiu et al. [90] has shown that increasing PCR cycle numbers directly increases the number of PCR products that contain sequences from two different DNA templates. Accumulation of these sequences, known as chimeras, is due to the creation of incompletely extended primers during the later cycles of PCR amplification that can act as primers for new DNA templates [91]. This issue is further exacerbated in marker gene sequencing studies due to the higher likelihood of homology between template DNA strands from the same or highly related species within a PCR [92].

Various strategies have been developed to reduce chimeric sequences in multiple template PCRs, with the use of high-fidelity polymerases with proof-reading capability being one of the most promising [93]. In 2019, Sze and Schloss [94] examined bias introduced through the use of different polymerases and found that significant differences in error rate and chimeric sequence generation could be found between them. Interestingly, they found that after 30 PCR cycles AccuPrime polymerases had the lowest chimera rate (0.9%), but the highest error rate (0.124%), indicating a strong trade-off between lowering error rate and chimera formation. They found that the Q5, KAPA, and Phusion polymerases had the lowest error rates (~0.06%), although the KAPA polymerase’s chimera rates were also the second lowest (2.3%) among polymerases tested. Based on these results, they recommended the use of KAPA polymerase for 16S rRNA gene sequencing studies. However, despite these differences they concluded that the choice of polymerase has little impact on the biological interpretations of community-wide measurements of diversity when using appropriate chimera filtering software such as UCHIME [95]. Similarly, in 2016, Gohl et al. [93] examined multiple polymerases and found that the use of high-fidelity polymerases resulted in fewer chimeric sequences. In summary, these two reports indicate that the choice of polymerase can affect both the error rate of sequences and the abundance of chimeras. To reduce these issues during PCR amplification, it has been suggested that researchers should use the highest possible fidelity polymerases and minimize the number of PCR cycles used [94].

Finally, one potential source of bias in PCR is the amount of template added to reactions. Although the overall goal would be to standardize the input DNA amounts across all community samples, achieving this goal is currently not possible for most microbiome samples. Due to the abovementioned extraction differences, copy-number variations between organisms differing between samples and, most importantly, the unquantified amounts of background “non-target” DNA that can be present in samples, it is quite difficult to assess the true “on-target” template amounts being added to PCRs. Given this, it is usually more productive to generate similarly strong amplification, regardless of template input differences, from as many samples as possible so that post-library normalization will generate as consistent sequencing between all samples as possible (i.e., will generate similar “sequencing effort”). In conclusion, researchers should keep a consistent protocol throughout their microbiome study and should report all PCR parameters within the methodology section of their report.

### Sequencing platform

The majority of DNA sequencing projects are now done on Illumina sequencing platforms, which generally show preferable DNA sequencing performance when compared to other short read technologies such as Roche 454 sequencing or Ion torrent sequencing [7]. In 2016, D’Amore et al. [96] compared several sequencing platforms using 16S rRNA gene sequencing to determine the amount of bias introduced by the choice of DNA sequencing platform. They found that each DNA sequencer had its own unique bias profile that caused significant differences in the observed community make up that explained small levels of variation between microbial profiles [96]. This confirms earlier work by Salipante et al. [97] who compared the Illumina MiSeq and Ion Torrent Personal Genome Machine and found that overall the disparities between the two platforms were minor. Accordingly, these studies show that, while the choice of DNA sequencing platform can impact the observed community, the systemic bias introduced tends to be smaller than sample-to-sample variation.

Recent advances in long-read sequencing technologies have now enabled the sequencing of large DNA fragments. These advancements have enabled full-length marker gene studies and more comprehensive evaluation of metagenomes [98, 99]. However, historically long-read technologies have been plagued by significantly higher error rates (5–15%) [100, 101], particularly when compared to short-read technologies such as Illumina sequencing (0.01–3%) [96]. Nonetheless, recent work by Schloss et al. [102] and Johnson et al. [98] has shown that advancements in PacBio circular consensus sequencing have led to observed error rates as low as 0.027%. Unfortunately, this comes at a significant cost to sequencing throughput and other long-read sequencing platforms such as the MinION still have significantly lower accuracies than its short-read competitors. While the throughput of long-read platforms are still significantly lower than Illumina short-read technologies, the use of full-length marker genes has been shown to provide considerable advantages [98]. These advantages include the elimination of systemic biases between studies that have sequenced different marker gene regions and improvement on our ability to resolve lower levels of taxonomy.

It should also be noted that advancements in DNA sequencing technology have introduced the use of linked reads and proximity ligation strategies to produce high-quality synthetic long reads [103]. Using these methods, it is possible to produce higher quality genome assemblies; however, users should be aware of the potential biases these methods introduce due to the various enzymes used during library preparation [104]. As such, researchers should be aware that data produced through other library preparation methods may not be directly comparable.

### Primer choice during marker gene studies

Despite the recent advances in long-read technology, marker gene sequencing is still predominately performed with high-throughput short-read technologies. These technologies, such as the Illumina MiSeq and HiSeq, are limited to short fragments ranging from ~150 to 550 bp. This has resulted in the creation of multiple different primer sets to target different variable regions on marker genes, such as the bacterial 16S rRNA gene [105–107]. Unfortunately, these primer sets differentially amplify different bacterial taxa, and no 16S rRNA gene primer set has been shown to equally amplify all bacteria. This is due to multiple reasons, including differing hybridization rates between taxonomic groups [7] and the presence of inhibitory flanking DNA [108], often caused by secondary structure and/or GC content differences. These unequal priming events lead to over- and under-detection of various bacterial groups, depending on the primer set used, leading to obstruction of the true underlying community composition and large taxonomic differences between studies that use different primer sets [109]. Numerous studies have compared the detection biases of various primers in different environments, and primer choice remains a debated topic within the field [20, 75, 106, 110, 111]. While large projects such as the Earth Microbiome Project recommend the usage of the V4 region, this choice does suffer from the underrepresentation of several important taxonomic groups, including *Actinobacteria* and *Propionibacterium*, while over representing groups such as *Streptococcus*, *Treponema*, and *Prevotella* [75, 107]. It is highly recommended that researchers use a primer set that is either directly comparable to previous literature or can detect the groups of bacteria they are interested in studying.

Fortunately, as mentioned previously, improvements in DNA sequencing technologies have now begun to enable higher throughput full-length marker gene sequencing. This has the promise to reduce biases between studies that use primer sets that target different regions on marker genes. However, the primers used in full length studies will still have different priming rates between taxonomic groups.

### Metagenomic library preparation

Currently, the majority of metagenomic shotgun sequencing projects rely on high-throughput short-read Illumina DNA sequencing [112]. Several different library construction methods have been developed for Illumina sequencers, which generally consist of three to four different stages: DNA fragmentation, optional repairing of DNA fragments, ligation of platform specific adaptors, and optional library PCR amplification [113]. Multiple different strategies have been used to accomplish these tasks including enzymatic digestion, mechanical shearing, and the use of transposons. In 2019, Sato et al. [113] compared the biases introduced by using different library preparation kits by examining both mock communities and isolated bacteria. They found that library preparation using the now-discontinued Illumina Nextera XT kit suffered from strong biases toward high-GC content regions. Examination of mock communities prepared using the XT kit resulted in the under-observation of low-GC content species such as *Staphylococcus aureus*, *Brachyspira pilosicoli*, and *Streptobacillus moniliformis*. Further investigation also revealed that minor biases toward high-GC content were also present in library preparations using mechanical fragmentation, likely due to non-random DNA shearing during sonication methods [114]; however, these biases were smaller than in the case of XT kits. Interestingly, the other library preparation kits they tested, including the new Nextera Flex (replacement for XT) Illumina transposon-based method, showed relatively little bias when compared to one another. Overall, this indicates that the observed microbial community can be systemically biased when using library preparation methods such as the Illumina Nextera XT kit or sonication-based mechanical fragmentation.

## Marker gene bioinformatics

After sequencing has been accomplished, a significant amount of bias can be introduced during downstream bioinformatic analysis [55]. A typical analysis begins with quality filtering to remove reads with ambiguous or error-prone base calls. Despite this step removing a significant amount of poor-quality data, it has been shown to only introduce a relatively small amount of bias directed toward the detection of low-abundance microorganisms [8]. After quality filtering, reads must be assigned to an analytical unit using either error-correction denoising algorithms or operational taxonomic unit (OTU) picking.

### Taxonomic units

As both sequencing and PCR can introduce base errors during sequencing experiments, it is not recommended to analyze sequences directly. Instead, users can choose to either cluster sequences together into OTUs or correct sequencing errors using denoising algorithms. The choice to use denoising algorithms or OTU-picking strategies has been shown to create systematic bias in marker gene sequencing studies [8, 115]. Within each of these distinct categories, further systemic bias can be introduced based on the implementation of the chosen denoising or OTU-picking algorithm [7]. In the case of OTU picking, there are three major methods for defining OTUs: reference-based, de novo, and open-reference clustering. During reference-based clustering, sequences are compared to known marker genes within a database and clustered at a specific percent identity cutoff [116]. This differs from de novo clustering strategies as these methods do not rely on reference databases, but rather cluster all sequences within a study based on pairwise nucleotide distances [117]. Finally, open-reference clustering uses both strategies by first performing reference-based OTU picking and then clustering the remaining sequences in a de novo manner [118]. Overall, the clustering of sequences in these manners allows researchers to mitigate the impact of sequencing errors on any one read.

Currently there is mixed evidence on which strategy is best when attempting to define OTUs [7]. While de novo methods have been shown to create higher quality OTU classifications [117], they often suffer from being unstable both within studies [119] and between studies [120]. Furthermore, while the use of different strategies can result in significantly different richness counts [117], they often resulted in similar beta-diversity metrics, indicating that, while biases are present, they are often smaller than sample-to-sample variation [121]. Overall, it currently remains unclear which clustering strategy reveals observations closest to the true community, although recent work by Edgar [122] has shown that, regardless of clustering strategy, the use of OTUs often inflates the diversity and richness present within a microbial community.

To help counteract these issues, various denoising algorithms have been created to resolve single-nucleotide accuracy while also generating sequences that can be compared between studies [120]. Currently, there are several denoising algorithms that generate amplicon sequence variants (ASVs), including DADA2 [123], Deblur [124], and UNOISE [125]. While the goal of these three tools is similar, they achieve it through different mechanisms. Deblur first filters reads for possible artifacts through the alignment of reads against sequence artifact databases and the removal of reads that do not map to the 88% OTU Greengenes database. It then aligns all remaining sequences against one another and uses information on their abundances and the error rate of Illumina sequencers to remove sequences derived from errors. UNOISE uses a similar approach that first clusters sequences together and then uses pre-set parameters to remove error-derived sequences. Unlike the other two methods, DADA2 generates a parametric error model for each sequencing run and then uses quality score information to remove sequencing errors.

These algorithms have been compared to standard OTU-clustering methods and in many cases have shown preferable results. Nearing et al. [8] found that while open reference-based OTU clustering resulted in the inflated richness of mock communities, all three denoising algorithms had richness scores comparable to the expected number of biological sequences. They also found that all three denoising algorithms resulted in comparable biological conclusions when examining metrics that were weighted by abundance. Subsequently, Caruso et al. [115] have shown that denoising algorithms outperform conventional de novo OTU-clustering strategies on several different mock communities. It should be noted that denoising tools have been developed using mock communities and as such their pre-set parameters and modeling behaviors may be overfit toward simple microbial communities. This makes it extremely important to be consistent in your choice of algorithm when comparing data within and across studies. However, overall, the data does suggest that denoising algorithms may be a preferable option for analytic unit assignment in future marker gene studies.

Regardless of the advantages and disadvantages of each algorithm, it is important to record which strategy was used to assign analytical units during analysis. Due to the intrinsic nature of each algorithm, they all generate their own systemic biases which become especially apparent during the examination of rare taxonomic groups [8].

### Taxonomic classification

Taxonomic classification of ASVs or OTUs poses a second challenge in the bioinformatic analysis of microbiome sequencing data. There have been multiple different strategies used to assign taxonomy to ASVs or OTUs throughout the years [126]. Common strategies include the use of kmer-based classifiers, such as naïve Bayes or Kraken2 [127, 128], local alignment search tools [129], or multiple global alignment strategies [130]. Each of these classifiers have been shown to have varying accuracy scores depending on the sample that is being analyzed [126, 128, 131] and introduce systemic biases in their ability to resolve the classification of novel taxonomic groups [131]. Furthermore, each of these methods is dependent on the use of reference databases that can result in additional differences between studies.

Currently, there are four commonly used marker gene databases for microbiome studies: Greengenes [132], SILVA [133], Ribosomal Database Project (RDP) [134], and NCBI [135, 136]. Interestingly, each of these databases use their own method for taxonomic classification assignment. While all four use seven main ranks: domain, phylum, class, order, family, and genus, both the SILVA and RDP databases also contain intermediate ranks. Furthermore, unlike the other three databases that use manually curated systematics to assign taxonomy, Greengenes taxonomy is assigned based on de novo 16S rRNA gene tree construction [137]. While work has shown that Greengenes, SILVA, and RDP can be mapped onto NCBI taxonomy with only low levels of dissimilarity, there are many instances where Greengenes, SILVA, and RDP cannot be mapped reliably to one another [137]. This distinction is important as slight differences in taxonomic assignments between studies can lead to substantial differences in interpretation of the biological conclusions from studies.

### Copy number correction

Unfortunately, not all marker genes that are studied are present as single copies within microorganisms. The commonly sequenced 16S rRNA gene is present in variable numbers among both bacteria and archaea [138]. While it has been shown that 16S rRNA gene copy number rarely varies within species, it often increases in variation with taxonomic distance [139]. Furthermore, due to these differences, 16S rRNA gene sequencing experiments can be biased toward the detection of bacteria with higher 16S rRNA copy numbers [140]. While this bias will have minimal impact on between-study comparisons, they can severely distort the observed composition from the underlying truth [141].

Work by both Kembel et al. [140] and Angly et al. [142] has found that 16S rRNA gene copy number variation exhibits strong phylogenetic signals. This has led to the development of multiple different phylogenetic methods for correcting 16S rRNA gene copy number variation within microbiome studies. Louca et al. [141] recently compared three different tools (PICRUSt [143], CopyRighter [142], and PAPRICA [144]) in their ability to correctly predict the number of 16S rRNA gene copies within a wide range of both bacterial and archaeal clades. In their analysis, they found that independently of the tool used, 16S rRNA gene copy number could only be accurately predicted for a small number of tested genomes that had low levels of sequence divergence from reference 16S rRNA genes [141]. This indicates that while copy number correction methods could be useful in well-studied environments, they generally introduce more noise than they correct. Fortunately, like most bioinformatic methods that rely on reference databases, their accuracy may increase as new genomes are sequenced.

## Metagenomic shotgun bioinformatics

Unlike marker-gene sequencing, metagenomic shotgun sequencing involves the sequencing of all the DNA contained within a sample. This can significantly complicate bioinformatic workflows, and, as a result, two main approaches for data analysis are available: referenced-based metagenomics and de novo metagenomic assembly. Each of these methods have their own benefits and detriments and often analysis with one method can complement the other.

### Reference based

Reference-based metagenomic shotgun sequencing strategies generally involve three different approaches by comparing sequenced DNA to databases containing reference genes, marker genes, or translated protein sequences [145]. Multiple tools exist for each strategy; however, they often are either comparing full-length sequences, marker gene sequences, or kmer sequence composition to reference databases to achieve taxonomic and functional assignment [146]. McIntyre et al. [147] compared 11 different metagenomic classifiers and found that the number of species identified within the same sample could differ by three orders of magnitude depending on tool choice. However, they found that the overlap between the true taxa within the sample and most abundant species identified was relatively high, indicating that filtering of low-proportion species could improve accuracy and reduce the number of false positives. Furthermore, they found that the most commonly identified false positives belong to the phyla Proteobacteria, Firmicutes, or Actinobacteria. More recent work by Ye et al. [145] compared 20 different metagenomic classifiers and found that all tools had relatively high area under the curve for precision and recall with the exception of the phylogenetic marker-gene-based tools MetaPhlAn2 [148] and mOTUs2 [149]. The authors also found that DNA-based strategies, in particular Kraken [150] and taxMaps [151], tended to have abundance estimates that were closer to the underlying truth than other strategies. This is most likely due to protein classifiers lacking untranslated regions within their database and marker-gene strategies lacking marker genes for specific taxonomic lineages. However, it was also found that DNA strategies tended to suffer from large numbers of low-abundance false positives while marker gene strategies did not [145]. Overall, these studies indicate that the use of DNA-based strategies with an appropriate abundance filter, such as Kraken and its related tools Kraken2 [152] and Bracken [153], is recommended due to their favorable performance metrics. However, it should be noted that a combined approach using marker gene-based classifiers may be required when researchers are interested in studying low-abundance taxa.

Finally, it was shown that all classifiers underperformed as the number of poorly described taxonomic groups increased within a sample [145]. This issue has become especially apparent within the study of the human virome, where sequence databases cover only a small amount of total viral diversity [154, 155]. This issue highlights one of the largest biases in reference-based approaches as they are only able to classify functions and taxonomic groups within the target reference database. This limitation suggests that additional analysis on poorly described samples using de novo assembly could significantly improve insights into the observed underlying microbial composition.

### Metagenomic assembly

Unlike reference-based metagenomics, de novo assembly methods do not rely on the use of existing reference sequences. Instead, they take advantage of sequence overlaps between DNA reads to generate longer predicted sequences known as contigs and scaffolds [112]. This process is known as assembly and can be accomplished by multiple different programs including the popular tools MEGAHIT [156] and metaSPAdes [157]. The recovery of complete genomes from assembly is often not possible due to their inability to resolve highly repetitive regions [158] and regions of similarity between different genomes [112]. This has led to the development of a second step known as binning. Binning takes advantage of multiple features, including the co-abundance and coverage of contigs between samples [159] and the grouping of contigs with similar kmer frequencies and GC content [160, 161]. This allows contigs to be grouped into bins which represent groups of contigs likely co-occurring within the same genome.

This process has enabled the recent discovery of thousands of novel metagenome-assembled genomes in human microbiome samples [162–164]. However, while assembly methods have a lower dependency on reference databases, they are biased in multiple other aspects. Due to the complex composition of microbiome samples, assemblers are often not able to determine the relationship between a large portion of reads within a sample [112]. This can result in the under classification of functional genes such as rRNA genes that are often found in repetitive regions [158]. Vollmers et al. [165] recently compared various tools for assembling metagenomes and found significant biases in their ability to identify both high- and low-abundance organisms. Similarly, work by Yue et al. [166] compared various binning strategies and found that tools output differing results, with DASTool, MetaWrap, and MetaBat2 showing the strongest results. This indicates that, like reference-based approaches, the choice of bioinformatic software could have significant impacts on the observed results.

Finally, like reference-based metagenomics, the annotation of assembled bins and genomes is dependent on known reference databases. Several reference databases exist for this purpose including NCBI RefSeq [167] and the Genome Taxonomy Database [168]. Currently, there is debate within the field as to which databases provide the most accurate representation of the underlying data. This can result in biased annotations of genes or taxa that are overrepresented within a database which can considerably bias the information obtained during analysis [169]. Despite these drawbacks, assembly based methods offer a complimentary strategy to reference-based approaches that offer the ability to identify novel microorganisms within a sample.

## Abundance correction methods

### Bias adjustment using mock communities

In 2015, Brooks et al. [45] presented a novel protocol for identifying and correcting biases for specific taxa within a microbial community using mock community mixtures. Using 80 different mock communities of specific taxa, they were able to predict the true abundance of those taxa within sequenced samples of unknown composition. Based on their model, they found that biases could introduce abundance error rates as high as 85%, indicating the importance of incorporating them during downstream analysis. Furthermore, they found that the predicted true abundances of specific bacteria within clinical samples better reflected the physiology and diagnosis of patients. For example, they found that the predicted abundance of *G. vaginalis* using their modeling method correlated better with pH levels than the originally observed proportions.

Further work in later years by Krehenwinkel et al. [170] and Bell et al. [171] involved the use of simple linear regression models of observed proportions against actual abundances to estimate experimental biases. Like previous work, they found that each taxon within a sample had different levels of observed biases. While they were able to show strong model fits for many taxa, these studies suffered from their inability to consider the interactions between biases within a single sample, which is critical when modeling compositions using proportions [6].

Fortunately, recent work by McLaren et al. [6] has shown the promising ability to adjust biases introduced throughout microbiome sequencing experiments using a mathematical model based on the ratio of observed reads between different taxa. Within this model, they show that, unlike proportions, biases that impact ratios between taxa are independent of sample composition. This critical finding helps address previous issues with models that failed to consider interactions between various biases within samples. The authors then go on to show that, using a simple mathematical model based on taxon-specific multiplicative biases and the ratios between taxa, they could predict proportions that closely matched observed results from mock communities. Furthermore, they showed that the use of mock community calibration controls can help adjust observed proportions to their true values in samples with unknown composition.

Unfortunately, all these methods for bias adjustment rely on the ability to use mock communities as calibration controls. While this may be feasible for a small number of bacteria as suggested by Brooks et al. [45], it is currently not feasible for the majority of microbiome sequence-based experiments. With the inability to culture a significant proportion of microbes, many projects will suffer from the inability to create cell-based mock communities [172]. This will result in the inability to measure DNA extraction biases which have been shown to play a significant role in microbiome studies. Furthermore, due to the resolution of many 16S rRNA gene sequences, OTUs or ASVs of interest may not currently correspond with any known living organisms. This would again create barriers in the ability to collect and create mock communities. Despite these drawbacks, there are many instances where mock community construction is possible, as shown by Brooks et al. [45], and these methods should be considered when plausible. Furthermore, a publicly available database of extraction efficiencies of different microbes using different extraction protocols would substantially improve the usability of these methods in the future.

### Determining absolute microbial abundances

**Fig. 2:**
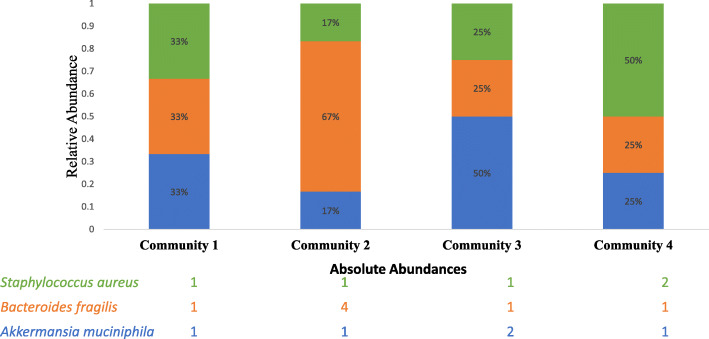
Microbiome data is compositional and does not represent absolute counts. As a consequence of the arbitrary total amount of sequencing reads a DNA sequencing platform can generate, the resulting data is compositional. This means that one DNA sequence does not correspond to one cellular unit, and an increase in relative abundance from one group can result in the decrease of relative abundance of another. This can occur even when the absolute abundance of a group does not change

Unlike the use of culturing, qPCR, or flow cytometry, DNA sequence-based microbiome studies currently lack the ability to determine absolute microbial abundances. This is due to the compositional nature of DNA sequencing data where one read does not correspond to one cellular unit [173]. This can often obscure the analysis of microbiome data, as the increased relative abundance of one taxon will always result in the decreased relative abundance of all other taxa in a sample, even if they have not actually changed (Fig. 2). In 2017, Vandeputte et al. [174] combined 16S rRNA gene sequencing data with flow cytometry and qPCR data to generate quantitative microbiome profiles (QMP). While this method allowed the authors to generate new insights into the microbial etiology of Crohn’s disease, it suffers from multiple different downfalls. To generate QMP data from 16S rRNA gene sequencing data, the number of 16S rRNA gene copies in each cell must be determined. Unfortunately, this process is unreliable for most bacterial and archaeal genomes [141]. Furthermore, QMPs cannot consider multiple different biases that are introduced throughout sequencing experiments that disrupt the relationship between cellular counts and sequence abundance [6, 45]. This includes differing DNA extraction efficiencies and PCR amplification efficiencies between different taxonomic groups. Decades of work in “classical” environmental microbiology have also shown that it is not a trivial matter to obtain accurate cell counts for many samples. There are also the considerations of live vs. dead cells included in counts (or the accuracy of live-dead stains, if used) and the potentially variable biases (depending on sample type) introduced by the sample preparations required to enable microscopy or flow-cytometry counting. Even if accurate counts could finally be obtained, the end-point nature of current high-throughput sequencing PCRs means that resolved abundances are “semi-quantitative” at best, as they are not based upon the same technique as qPCR.

Tkacz et al. [175] presented a second method in 2018 for determining absolute microbial abundances from DNA sequencing data. They proposed the use of synthetic chimeric DNA with known quantities as controls during marker gene sequencing experiments. Furthermore, they suggest that these chimeric DNA sequences can be modeled after different taxonomic sequence signatures to control for amplification biases. This provides a significant improvement over the previous attempts; however, it still suffers from differences in marker gene copy numbers and differing DNA extraction efficiencies.

## Methodology standardization and reporting

### Consistent study protocols

A significant portion of the bias between microbiome studies is introduced through the use of different protocols [53, 176]. This has led to attempts to standardize protocols within the microbiome field to allow for improved inter-study comparisons. In 2010, the Earth Microbiome Project [176] was initiated as an attempt to collect and sequence microbiome samples from around the world. With the initiation of this project, several standardized protocols were developed including the acquisition of metadata, DNA extraction, and both marker-gene and shotgun-sequencing library preparation. Following this, the International Human Microbiome Standards group compared a number of different protocols on human fecal samples and presented recommended protocols based on reproducibility and accuracy of community diversity [53]. While following these recommended protocols would reduce biases between studies, not all protocols are appropriate for the diverse number of biological questions at hand [6]. For example, some protocols may need to be altered if the researchers are interested in either the live or total microbial community contained within a sample. Furthermore, the use of standardized protocols still does not solve the systemic biases introduced during sequencing experiments that led to distorted observations of the true underlying microbial community.

Despite these issues, it is still recommended that whenever possible, protocols between closely-related studies should be replicated to reduce the amount of bias introduced between each study. As previously mentioned, when possible, manufactured kits or the use of well-validated protocols such as those outlined by the International Human Microbiome Standards project or Earth Microbiome project should be used. Furthermore, the use of classification tools such as random-forest modeling or neural networks should be validated on data from more than one study group to ensure the reliability of their model on real world data. Overall, when interpreting results, researchers should remember that the observed communities are only one representation of the true underlying abundances contained within the sample.

### Methodology reporting

While there are several promising approaches to help reduce the amount of systematic bias within microbiome studies, the best way to help between study comparisons is the creation of a comprehensive methodology section. Not only will this allow researchers to replicate your results but will also allow them to help identify why results may not be reproducible from one study to another. A number of studies including projects such as the Earth Microbiome project and more recently the STORMS microbiome project [177] have addressed this issue. As such, we will only briefly outline in the section below as well as in Table 2 key information that should be present in the methodology of all microbiome studies.

**Table 2:** Key information to report within sequenced-based microbiome manuscripts

Processing step	Parameters
Sample collection	- - Description of samples of interest - Collection location - Collection device - Number of different collection personnel - Sterile techniques used - Use of technical replicates
Sample storage	- Media used to store samples - Length of storage and storage conditions - Length of time spent unpreserved
DNA extraction	- DNA extraction method - If using manufacturer kit: ◦ Product name ◦ Optional steps used - Any methods used to reduce or remove possible contaminants
Library preparation	- PCR parameters including: ◦ Polymerase ◦ Cycle number ◦ Thermal profile - Marker gene studies: ◦ Primer names and gene target(s) - Metagenomic shotgun studies: ◦ Library preparation kit name/protocol ◦ Detailed information about: ▪ DNA fragmentation ▪ DNA fragment repair ▪ Ligation ▪ PCR amplification - Normalization method - Amount of starting template used
Sequencing	- Sequencing platform - Consumable product names - Whether demultiplexing or any other sequence pre-processing on-instrument was done
Bioinformatics	- All tools used to process raw data along with: ◦ Goal of tool ◦ Version number ◦ Non-default parameters - Any database names along with their version numbers - Any statistical tests used to analysis data

All sample collection procedures should be included within the methods section. This includes the collection method and sterile techniques used during sample collection, as well as the media used to store the samples. Furthermore, authors are also recommended to give the general length of time samples remained in storage before processing began. One of the most critical pieces of information included in the methods section should be the DNA extraction method used during sample preparation. As highlighted, above DNA extraction is one of the largest factors that introduces systematic bias within a study. If a DNA extraction kit is used, author must report the manufacturer and product name, along with any modification or optional steps that the authors used, such as the addition of proteinase K. Within this section, it is also recommended that the authors report all steps taken to reduce any possible sources of contamination, such as the use of biological safety hoods and inclusion of blanks (critical for low-biomass samples) during DNA extraction.

Depending on the type of DNA sequencing used to investigate the samples of interest, the exact reporting parameters will differ between marker gene or metagenomic shotgun sequencing. Researchers should report all parameters used during PCR amplification, including the number of cycles and the type of polymerase used. The authors should also report the primer names and sequences if custom primers are used, during marker gene sequencing. If a commercial metagenomic library preparation kit was used, the authors should report the manufacturer as well as the product name and any modifications or optional steps used during sample processing. The amounts of template and the method of library normalization should be reported within the methods for both marker gene and metagenomic shotgun sequencing studies. The DNA sequencing instruments, along with which consumable versions used during sequencing, should be reported.

No matter what approach is used to investigate your samples, the bioinformatics method section of a manuscript should include the names and versions of all bioinformatic tools used within the workflow. This includes any databases that were used for classification throughout the study and all non-default parameters used. Any programs used for statistical analysis should be reported, along with any hypothesis tests that were used to investigate the samples of interest. Finally, all code along with its documentation and comments should be deposited in a public repository (e.g., GitHub) and linked within the methodology section of its accompanying study.

## Closing remarks

The study of the human microbiome has clearly created a better understanding of the relationship between humans and microbes. However, multiple different experimental steps have led to the inclusion of significant biases within the human microbiome literature. In many cases, this has led to the inability to compare results from one study to another. The use of consistent protocols on similar sample types could help address issues of between-study bias [7]. This will, however, require the development of further environment-specific protocols, as not every sample is amendable to currently recommended solutions. Furthermore, authors should report the key parameters outlined within this review in all microbiome-based sequence studies to help ensure the reproducibility of their results.

While reporting these key parameters will help with addressing between-study biases, it will not help address within-study biases that can lead to distorted observations of the true underlying microbial composition. These issues generally stem from multiple areas, including the inability to quantify extraction efficiencies between different microbes and different DNA amplification rates. Without solving these issues, the true view of the absolute abundances of microbes within the human microbiome will not be possible. This is critically important to understand when evaluating methods that rely on qPCR, flow cytometry, or DNA spike-in methods. Although recent work in the field of mathematical modeling shows the promising ability to adjust most biases within a microbiome experiment, they currently rely on the ability to create mock communities [6]. Unfortunately, as stated previously, this is currently not possible for many microbes. Despite this, we suggest that future work should attempt to quantify DNA extraction efficiencies for various protocols and health-related microbes as it is currently one of the largest areas of bias within microbiome studies.

Overall, it is currently recommended that all methods in a study should be kept consistent and, when comparing to previous work, the same protocols should be followed. If this is not possible, protocols should be benchmarked between studies to identify whether consistent patterns of bias can be found between them. This will give researchers additional information that can be used when comparing results between studies. Finally, all biological conclusions generated from sequenced-based microbiome experiments should be validated through other molecular techniques to ensure that the underlying results are robust to biases introduced during microbiome sequencing experiments.

## Data Availability

Not applicable.

## Abbreviations

MGS: Metagenomic shotgun sequencing
OTU: Operational taxonomic unit
ASVs: Amplicon sequence variants
PMA: Propidium monoazide
RDP: Ribosomal database project
QMP: Quantitative microbiome profile
PCR: Polymerase chain reaction
qPCR: Quantitative polymerase chain reaction
